# Health-Oriented Self- and Employee Leadership in Virtual Teams: A Qualitative Study with Virtual Leaders

**DOI:** 10.3390/ijerph17186519

**Published:** 2020-09-07

**Authors:** Ilona Efimov, Volker Harth, Stefanie Mache

**Affiliations:** Institute for Occupational and Maritime Medicine, University Medical Center Hamburg-Eppendorf, Seewartenstr. 10, 20459 Hamburg, Germany; harth@uke.de (V.H.); s.mache@uke.de (S.M.)

**Keywords:** health-oriented leadership, virtual teams, virtual leadership, leadership behavior, factors of influence

## Abstract

Virtual teamwork as a new way of working is becoming increasingly prevalent in a growingly globalized and digitalized working environment. Due to the associated raise in health-related stress factors at the workplace and the central role of leaders in workplace health promotion, the aim of this study is to obtain initial findings on the use of health-oriented self- and employee leadership in virtual teams from the perspective of virtual leaders. Semi-structured telephone interviews were conducted with 13 virtual leaders by using the problem-centered interview method. The collected data were deductively and inductively evaluated and interpreted using the qualitative content analysis according to Mayring. The results show that virtual leaders ascribed great value of health and showed great awareness in health-oriented self- and employee leadership. Physical activity and boundary management were particularly mentioned as health-oriented self-leadership behaviors. The majority of leaders described communication, building trust, support in boundary management and implementation of personal meetings as health-oriented employee leadership behaviors. In addition to social, technical, and personal factors, primarily organizational factors were mentioned as factors of influence in this context. For a more comprehensive understanding of health-oriented leadership, the inclusion of virtual team members in further research studies is necessary.

## 1. Introduction

The current working environment is constantly changing due to digitalization, automation, and individualization. Digital information and communication technologies (ICT) are increasingly being used and fixed working hours and locations are being dissolved [1]. Therefore, “New Ways of Working” (NWW) are emerging [2]. These NWW are manifold and must always be considered in an organizational context [3]. Among forms of flexible work, Kelliher and Anderson [4] include reduced working hours, non-standard or compressed working hours and various forms of remote working, such as e.g., virtual teamwork. Virtual teams can be defined according to three aspects: Virtual teams work in the same way as face-to-face teams over a certain period of time on common tasks and objectives, but their members are geographically distant from each other, sometimes with time differences, and communicate with each other mainly via ICT [5,6,7,8]. Virtual teams are on a continuum of virtuality, as ICT is used in different forms and intensities [7,9]. The widespread use and increasing demand for virtual teamwork in organizations worldwide illustrates the relevance of this new form of collaboration for research and practice [10,11,12,13].

### 1.1. Virtual Leadership

The characteristics of a virtual work context change not only teamwork [5], but also pose special challenges for virtual leaders [14]. Virtual leadership should not be understood as a leadership style, but rather as specific contextual conditions of leadership [15]. Leadership over geographical distance in flexible working conditions therefore requires specific skills from leaders: e-communication skills, e-social skills, e-team building skills, e-change management skills, e-technological skills, and e-trustworthiness [16]. It has been shown that classic leadership concepts applied in traditional team structures cannot simply be transferred to virtual leadership [17,18]. For example, hierarchical leadership appears to be difficult to implement in virtual teams [18]. Moreover, studies on transformational leadership in virtual teams show inconsistent results regarding effectiveness [6,19,20,21,22,23]. According to the current state of research, delegative leadership concepts that focus on autonomy, participation, and self-control are recommended for virtual teamwork [15,17,24,25]. Studies on shared leadership in virtual teams report positive influences on team effectiveness and performance [6,15,18,26,27,28,29,30]. Furthermore, it can be observed that virtual teams often work in a self-organized manner and competent team members emerge as leaders over the course of the project [26,28,31].

Generally, virtual teams are more difficult to lead than face-to-face teams characterized by presence and direct interaction. Virtual leaders thus face increased work demands and special challenges, especially with regard to building and maintaining trust [6,10,15,25,26,31,32,33,34]. Little or no direct contact due to geographical distance and time differences may lead to a loss of motivation, difficulties in building team cohesion, and challenges in team coordination [15,19,35]. Communication via ICT in virtual teams is a crucial challenge for leaders as technical problems can hinder effective and smooth communication [11,35] and non-verbal elements of communication can get lost [35]. Different language skills [36] and cultural influences can make mutual understanding and building personal relationships even more difficult [24,32,35]. In addition, greater attention must be also paid to the health of employees in a flexible working environment [17]. In this way, the specific working conditions of virtual leaders can be experienced as psychological stress factors and thus potentially affect the health of leaders [17,37,38].

To date, there are not enough empirical studies to gain valid insights into the function of virtual leaders and the effectiveness of different virtual leadership styles. The influence of virtual leadership via ICT on the health of geographically dispersed team members is also completely unknown. Accordingly, there is still need for research in the field of virtual leadership [39,40], as e.g., virtual teamwork requires adapted leadership strategies [41].

### 1.2. Relevance of Leaders’ Health and Wellbeing

Psychosocial risk factors are the most frequently named health risks at workplaces in Europe [42]. In Germany, mental and behavioral disorders made up the second largest cost factor [43] and were also the most prevalent cause of early retirement due to illness [44]. Given the immense relevance of employee health for the economy and society, it is appropriate to take a closer look at occupational groups at higher risk. Due to their specific working conditions, leaders are exposed to an increased risk of potential stress factors and related health complaints [45]. Although numerous studies have proven the influence of leadership on the health, wellbeing, and performance of employees [46,47,48,49,50,51,52], the health of leaders themselves is less frequently investigated [14].

On the one hand, previous studies indicate positive health conditions for leaders by reporting fewer psychosomatic complaints, a higher sense of well-being, and more self-esteem than employees without management responsibility [53]. The positive study results may also be related to the special resources of leaders: Leaders report greater job autonomy and the ability to plan and organize their work independently [54]. On the other hand, further studies emphasize health risk factors associated with the workplace of leaders. Leaders experience psychovegetative complaints and exhaustion more frequently, show higher values for stress increase and for professional and quantitative overload and are exposed to higher work demands than their colleagues without managerial responsibility [54]. Thus, leaders feel a strong pressure due to deadlines, time, and performance, are exposed to disturbances and interruptions at work, and at the same time deal with various tasks [45,55]. An increase in leadership span is accompanied by a more frequent mention of these work demands [45]. In addition, a higher proportion of leaders than of employees without managerial responsibility report frequent work on Saturdays, Sundays, and public holidays [54]. A working time survey showed that leaders (28%) report more frequently work-related increased availability than employees (19%) [56]. The more employees the leader manages, the more the expected availability and actual contacting increases [56]. The extension of working hours and the work-related extended availability are often carried out on leaders’ own initiative. However, both phenomena are associated with negative effects on the health and well-being [54,57]. Leaders are not necessarily less likely to be ill than employees, but rather tend to work while being sick more often [55,58]. Thus, in addition to sick leave as a common indicator of employee health, presenteeism, i.e., the presence of employees at work despite illness, must also be taken into account [55]. About half of the leaders surveyed in the SOEP (German Socio-Economic Panel) Survey 2011 found it difficult to switch off from work [59]. They still think about work in the evening and about professional problems when they wake up, which leads to a so-called negative spill-over of work stress [59]. The phenomenon of interested self-endangerment describes a working behavior oriented towards professional success that endangers one’s own health. In particular, leaders with flexible working hours are more likely to work in their free time and despite illness [60].

Overall, leaders play a crucial role in workplace health prevention due to their role model function and evident influence on employee health [45,61,62]. A current meta-analysis emphasizes the relevance of leader’s well-being for the entire organization, as it affects leadership behavior and consequently the well-being and performance of employees [52]. Therefore, leaders themselves need to be in an optimal stress-strain situation, be aware of occupational psychological relationships between characteristics of work activity and human psyche, and need to realize possibilities for a positive health culture at the workplace [58,63]. Virtual leaders are exposed to special strains at their workplace, yet there is little research on their own health situation. There is therefore a need for research on their health status and their health-oriented self-leadership as well as employee leadership.

### 1.3. Health-Oriented Leadership

For this study, we applied the health-oriented leadership (HoL) concept according to Franke et al. [64], as it combines follower-directed and self-directed health-oriented leadership. All three components (SelfCare leader, StaffCare, and SelfCare follower) consist of three dimensions: value, awareness, and behavior. The SelfCare of the leader forms the basis of the HoL concept. In order to be able to lead oneself in a health-oriented manner, one’s own health must be classified as important (value), one has to be aware of one’s own health status (awareness), and actively care for one’s own health (behavior). Only if the leader can lead himself/herself in a health-oriented way, a health-oriented employee leadership is possible. It is assumed that a pronounced SelfCare of the leader will enable him/her to serve as a role model for the SelfCare of team members. According to the HoL concept, positive values for all three components together (SelfCare leader, StaffCare, and SelfCare follower) increase the well-being and health of the workforce and consequently reduce stress and health complaints [64].

Previous studies have been able to show different mechanisms of action of health-oriented leadership on employee health. On the one hand, leaders can directly influence employees’ health via their health-oriented employee leadership behavior [64,65,66,67]. Among the most effective behaviors on how to deal with exhausted employees are the (re)design of tasks, emotional support, communication of expectations and offers of help by the leader [68]. On the other hand, they can indirectly influence employees’ health via their own state of health [69] and their health-oriented self-leadership [62,64,67,70,71]. By showing self-directed health awareness and behavior, they can serve as role models for the health-oriented self-leadership of employees [64,70]. The combination of a pronounced health-oriented employee and self-leadership of both leaders and employees as well as low levels of hazardous health behavior correlates positively with less stress and better employee health [62]. Furthermore, health-oriented leadership does not only lead to better health status but also to concrete health-oriented behavior of employees, namely the participation in courses of workplace health promotion [71]. An international longitudinal study also demonstrated a direct and indirect influence of health-oriented leadership on health [63]. The survey results showed that health-oriented leadership has the greatest predictive power in explaining a sustainable improvement in several health characteristics. In the long term, it is also shown that health-oriented leadership leads to an improvement in team climate and an increase in work commitment. In addition, interaction effects between the perception of health-oriented leadership and strains and resources at the workplace were found. Leaders thus indirectly influence employee health by designing working conditions. It is argued that this leadership approach is crucial to enable job autonomy and participation at work [63]. All previous empirical studies on health-oriented leadership mainly examined the influence of leaders on the health and well-being of employees. So far only one interview study indicated that virtual leaders themselves are exposed to various psychological strains at the workplace, e.g., time and performance pressure, requirements that cannot be met, unforeseen challenges, and heteronomy [72]. Their specially developed compensatory activities served primarily to compensate psychological stress. Leaders surveyed differed in terms of their self-image in their leadership role, their perceived responsibility for accident prevention, their understanding of workplace health promotion and their experience regarding health promotion at work [72].

### 1.4. Study Aim

To date, there is a lack of empirical evidence on whether virtual leaders are aware of the health of their team members and whether they can perceive changes in the state of health of their team members despite geographical distances and digital, sometimes time-shifted, communication. Furthermore, it is unclear whether and in which ways leaders can directly influence the health of their team members, even though they may rarely or never see each other personally. Since no empirical study has yet examined the HoL instrument in the virtual work context, there are no scientific findings on the applicability or effectiveness of health-oriented leadership in virtual teams [17]. Due to the high emergence of virtual teams in an increasingly globalized and digitalized world [13] and the associated increase in health-related stress factors at work [38], there is much need for research to empirically investigate how health-oriented leadership is applied in virtual teams. Therefore, the study aim is to test whether the HoL concept (SelfCare of the leader, StaffCare) according to Franke et al. [64] can be applied to virtual leadership on the basis of qualitative interviews with leaders of virtual teams. This study adds first empirical insights on the health awareness and health-oriented behavior of virtual leaders and identifies influencing factors regarding the application of health-oriented leadership.

## 2. Materials and Methods

### 2.1. Participants

The study sample comprised 13 male leaders of virtual teams in Germany. Due to the specific working conditions of our sample, recruitment was carried out according to the snowball system via personal professional contacts and professional network platforms. Applying the inclusion criteria, 14 interviews were conducted with (1) adult (2) German-speaking virtual leaders (3) who had been leading a virtual team for at least a year, (4) who spend more than 25% of their working time communicating virtually and (5) who were working in IT, manufacturing, aerospace, or logistics industries. Based on a representative international survey on virtual teamwork [13], it was assumed that virtual teamwork is increasingly taking place in these industries in Germany as well. One interview conducted had to be excluded from the sample because leadership experience could not be confirmed during the course of the interview. Participation in this study was voluntary. Prior to the interview, all participants received written information about the study and signed an informed consent regarding the performance and recording of the interview. No personal relationship had previously existed with any interview partner. The study was conducted in accordance with the Declaration of Helsinki and approved by the Ethics Committee of the University Medical Center Hamburg-Eppendorf, Germany (LPEK-0051).

### 2.2. Procedure

In this exploratory study, 13 semi-structured, guideline-based telephone interviews were conducted in German with virtual leaders by using the problem-centered interview method (PCI) according to Witzel [73,74]. The qualitative research approach was considered to be the most appropriate for obtaining initial findings in a new field of research. The reasons for conducting interviews by telephone are based on research economics, flexibility in time and place as well as geographical expansion of the sample. The interviews were conducted at flexible times and either from company office or home office. The PCI method was chosen as it is used to record specific behavior, experiences, reasons, evaluations, and subjective opinions in a dialogue and aims at a common understanding process between the interviewer and the interviewee [74,75]. All four PCI instruments were used: a short questionnaire for socio-demographic data, a tape recorder, an interview guideline, and a postscript [73]. A pre-test prior to the interviews was conducted by the first author in order to receive feedback on the guideline. The contents of the interview guideline are illustrated in Table 1. The questions regarding the value of health, health awareness, and behavior are based on item examples of the HoL instrument (Appendix A) [64,76].

The first author introduced herself at the beginning of the interviews and created postscripts directly after the interviews. Only in one interview was the child of the interviewee present in the room—no other persons were present in any other interviews. No interview was repeated. In total, the interviews lasted between 21 and 88 min. The repetition of experiences and attitudes in the interviews revealed a theoretical saturation of this study. With regard to data analysis, all audio recordings from the interviews were anonymized and transcribed in terms of content and semantics [77]. The transcripts were deductively and inductively analyzed and interpreted using the qualitative content analysis according to Mayring [78]. Our used coding tree corresponds to the process model of content structuring [78]. For both analysis steps the software MAXQDA Plus for Qualitative Data Analysis (Version 20.0.6, 2020, VERBI GmbH, Berlin, Germany) was used. The data transcription and evaluation was carried out without involving the interviewees. All steps—from conducting interviews to transcription and data analysis—were carried out by the first author, a female psychologist (M.Sc.), as part of her master thesis. The first author had practical experience in qualitative interviewing at the time of the study. Supplementary Materials, the COREQ checklist (Consolidated criteria for reporting qualitative research) was used to ensure the quality of reporting on the methodology of this qualitative study [79].

## 3. Results

All leaders of this sample were male and aged between 32 and 58 years (mean age of 44.2 years, Table 2). Of the 13 interviewees, eight leaders (61.5%) worked in the IT sector, two (15.4%) in the manufacturing industry, two (15.4%) in the aerospace industry, and one (7.7%) in the logistics industry. Six leaders (46.2%) worked in medium-sized companies (50–249 employees), seven (53.9%) in large companies (>249 employees). All participants in the study were in permanent employment, and the majority worked full-time (84.6%). The interviewees worked mainly in company offices (61.5%) or at fixed workplaces at home (38.5%). In terms of leadership experience, the majority had been leading a virtual team for at least two years (61.5%), five leaders since one to two years (38.5%). Nine interviewees (69.2%) were leading small teams (<10 team members), two interviewees each (15.4%) were leading medium-sized teams (11–20 team members) and large teams (>20 team members). A total of five leaders (38.5%) were leading international dispersed teams. In all teams, face-to-face meetings took place at different frequencies. Due to the geographical distance of their team members, different ICT was being used. The most commonly used media included video conferencing systems, e-mail, and telephone.

### 3.1. Health-Oriented Self-Leadership

All leaders ascribed a high relevance to their own health. The high value of health was justified by the fact that good well-being and health also lead to better performance and balance at work in the long term. The majority of the interviewees assessed the current state of health positively. Only one leader reported a mediocre state of health due to personal reasons. The influence of flexible working conditions on the current state of health was evaluated differently. On the one hand, a positive influence was perceived, because the flexible working conditions enabled a self-determined work-life balance, which reduced stress and created well-being. Other leaders reported that working conditions or ICT had no influence on the state of health, because each person is responsible for his or her own health and can actively maintain it. On the other hand, the negative influence of working conditions on health was described by high levels of everyday stress, perceived pressure, increased speed of work and workload. One leader explained that his work close to the limit was being done out of self-interest:

“Personally, I tend to be close to my limit because I like that too, but I’m actually paying attention not to collapse. Even if the workload tends to be that way. So I try to take care of myself.”

(Employee #1, age 41–50 years, e-leadership experience 2–4 years).

Some leaders reported very high workloads in the past up to exceeding stress limits. Through these negative experiences, personal awareness towards one’s own state of health had changed permanently, and a health-oriented self-leadership had been learned, e.g., by keeping to working hours and a self-determined work-life balance.

“Well, I don’t like being sick. I’ve learned it the hard way, let’s put it this way. In my previous role I was (…) lucky to have people or one person in America. That means I was represented in Asia, Europe and America, which extended my working day and I was relatively close to burnout at some point. But I was, had no burnout, but that was not so far away. And that’s when I talked to my boss, that I can get out of projects and take care of my team. And I made some decisions for me, too.”

(Employee #6, age 41–50 years, e-leadership experience >4 years).

In general, all leaders stated that they are able to recognize stress situations and changes in their own state of health in time. Signs by which they perceived these changes were both physical and psychological symptoms as well as behavioral changes. Changes in their state of health were attributed to different causes. The majority of interviewees named work-related causes such as high intensity of work, psychological strain caused by one’s own manager, or personal failure to take breaks. On the other hand, some leaders attributed causes of change to their private situation. Several managers explained that a perception of health changes was learned in a gradual process over a long period of time. The duration of the learning process was based on personal attitudes and behavior:

“Firstly, pride, as one was extremely needed. And one did not want to give up this position so easily. (...) And then a certain degree of displacement mechanism “It’s not that bad.” (…) Of course one has collected successes during this time. So from 2009 to 2017 I expanded the technical department from 22 employees to over 80. (laughter) And the turnover and the company have also benefited from it. And there are of course successes, you might have to gain some distance to evaluate them from a different angle, and then set yourself different goals and priorities.”

(Employee #10, age >50 years, e-leadership experience 2–4 years).

A total of nine health-oriented self-leadership behaviors were identified in the interviews, which primarily served to compensate for psychological stress at the workplace (Table 3). The majority of leaders indicated physical activity and boundary management as health-oriented behavior. Especially flexible working conditions (e.g., extended working hours, different time zones) in virtual teamwork made it more difficult for leaders to switch off and recover from work. They separated work from leisure time by setting up their own rules regarding work-related extended accessibility, adherence to working hours and use of office equipment:

“I also set up rules for myself, so when am I reachable via all these channels that are available (…)? And I take great care to ensure that I am able to, let’s say, separate the two. Especially in view of home office. If I didn’t had any rules or didn’t set up any rules for myself, then I think this would have a negative effect on my health.”

(Employee #9, age 30–40 years, e-leadership experience <2 years).

Furthermore, several leaders described that sufficient sleep and a conscious diet were beneficial to their own health. However, recreational activities such as regular mindfulness training or daily self-reflection also helped to reduce stress and create awareness for one’s own body. In addition, leaders explained that social exchange with private or professional contacts helped to exchange information or obtain advice when under stress. Some leaders mentioned self-determined and flexible time management, use of occupational health services, or mental handling of stress as health-oriented self-leadership behavior.

### 3.2. Health-Oriented Employee Leadership

All leaders attached equal high value to the health of their team members and their own health. The concern for the health of their team members was explained by the employer’s duty of care and personal attitude as a leader. Some interviewees emphasized the value of health:

“Health is the most important thing to me and I also pay extreme attention to health and I am reflected upon every day and this is also my top priority for my employees. In the first place!”

(Employee #11, age >50 years, e-leadership experience >2 years).

Regarding the health awareness of leaders, the majority stated that they are able to detect changes in the state of health of team members even by geographical distance. This requires open communication, good social relations with team members and a personal claim to be able to identify health problems:

“I believe the primary goal is to have a trusting relationship. In fact (...) really collegial or even friendship-based. And I also had a colleague in the US (...), I knew then that his wife was divorcing him, so I virtually hugged him and had intensive conversations with him.”

(Employee #3, age >50 years, e-leadership experience 2–4 years).

Nevertheless, several leaders pointed out that changes in the health status of virtual team members are more difficult to detect due to physical distance. There are fewer opportunities to recognize signs of stress and the quality of communication is also lower compared to personal communication:

“I’m there every six to eight weeks, but within these six to eight weeks there is only virtual contact. And the personal contact is just different—when I look into someone’s eyes and see their whole behavior and voice—even in a video conference.”

(Employee #8, age 41–50 years, e-leadership experience <2 years).

Due to the aggravating conditions of virtual teamwork, increased requirements for virtual leadership were perceived. In addition, the understanding of leadership roles differed among some leaders. Thus, one leader felt a high level of responsibility towards his team members. Other leaders did not feel responsible for the health of their team members, but for creating healthy conditions:

“As an employer, I am not actually responsible for their health, but I am responsible for ensuring that my way of working and the job requirements do not have a lasting adverse effect on the health of my employees.”

(Employee #2, age 41–50 years, e-leadership experience 2–4 years).

In terms of health-oriented employee leadership, five behavior patterns were identified (Table 3). Almost all leaders explained that trust is the basic precondition for health-oriented employee leadership in virtual teams. Therefore, they set an example in their leadership style and communication and were taking the initiative in building trust. It was argued that trusting relationships in geographically dispersed teams enable team members to open up and report problems.

Communication was described by almost all leaders as a key health-oriented leadership behavior. A rich and asynchronous communication media appropriate to the purpose of communication was named as a basic principle. Discussions on health-related topics were conducted both in confidential individual discussions via (video) telephony or chat, but also together with all team members via virtual or face-to-face meetings. In this way, individual problems, state of health, or workload were discussed and supported. Within the team, joint decisions were made regarding communication media, working hours or opportunities for personal exchange. One leader explains that remote work environment encourages open communication via digital media in team meetings:

“And [the e-coffee time] is also used actively, so people really talk about private matters. And the fact that we really have this once a week creates the impression that people sometimes talk even more than when they sit together in their office (…) nobody is listening.”

(Employee #9, age 30–40 years, e-leadership experience <2 years).

Support in the boundary management of virtual team members was named as another health-oriented leadership behavior. In this respect, leaders paid attention to the extended accessibility and working hours of their team members. In order to facilitate the separation of working and leisure time for their team members, they actively communicated their expectations, jointly set up team-internal rules for extended accessibility, respected their team members’ boundary management behavior, and did not contact them outside of working hours.

More than half of the leaders reported that personal team meetings were initiated to facilitate virtual communication and team cohesion. Especially in geographically distant teams, personal meetings at the beginning of virtual teamwork were crucial in order to create a relationship of trust. Further team meetings in the course of the cooperation served to promote personal communication, joint solution of problems, informal exchange, and the communication of mutual appreciation. At personal team meetings, leaders observed team and personnel development, carried out team building measures and created health offers such as sports activities or expert lectures on health or team-related topics.

Lastly, several leaders described their delegation of decision-making authority and responsibility to virtual team members as health-oriented leadership behavior. In this context, they took on a supporting function and provided their team members with a lot of freedom of decision and action, participation, and autonomy. By creating a safe working environment and promoting autonomy, it was argued that as a result self-efficacy ultimately promotes the health of the employees. Due to the self-organized team structure of two teams, responsibility and leadership tasks are distributed either to several team members in different functions or to the entire team:

“I and we are convinced that a great deal of leadership and responsibility also lies with the teams. Accordingly, I believe it is very, very important to get people to take care of each other, or not to stop them from doing so, and to a certain extent to become a team, because that of course works much, much better than if someone had to keep an eye on it somehow and the colleagues simply see each other much, much more often than I do now.”

(Employee #4, age 30–40 years, e-leadership experience >4 years).

### 3.3. Factors of Influence

With regard to the application of health-oriented leadership, organizational factors in particular were identified by leaders as decisive influencing factors (Table 4). Thus, company management, flexible working conditions, an open company culture as well as structural offers such as occupational health offers, upgrade training courses, and social events were perceived as supporting. The greatest potential for change for a better application of health-oriented leadership was also seen in the organization. Management, on the contrary, has also been experienced as aggravating by setting unattainable goals, very high expectations for the performance and readiness of employees and ignoring health at work. The lack of structural offers or aggravating working conditions were also experienced negatively in the application of health-oriented leadership: these include large time differences in international teams, a high workload, increasing extended accessibility, and a merging of work equipment.

In addition, social and technical factors were perceived as both supporting and aggravating factors. On the one hand, team members were seen as an important support for team processes and team development by paying attention to interpersonal problems or communication difficulties. Since virtual leaders are not able to lead team members in personal contact, self-organized teamwork or mutual support by the entire team was perceived supportive. In addition, other leaders in the company were reported to act as role models for one another. On the other hand, our interviewees stated that virtual cooperation is complicated by communication difficulties. Misunderstandings, among other things due to predominantly written communication and insufficient language skills or cultural differences may lead to differences in cooperation. Furthermore, an unhealthy leadership style of one’s own manager was described by one interviewee as another aggravating factor. Digital media was seen as supportive as it allows flexibility in working conditions and has a positive effect on the ability to work. Yet technical failures or problems may lead to communication difficulties or stimulus satiation and cause stress. Finally, personal factors such as one’s own ambition as well as too high demands and expectations of oneself were also experienced as aggravating factors in health-oriented leadership.

## 4. Discussion

To our knowledge, this study is the first to provide empirical results on health-oriented leadership of virtual leaders. In our interviews, German virtual leaders were asked about the value of health, their health awareness and behavior in terms of health-oriented self- and employee leadership. Referring back to the HoL concept [64] and its proposed heuristic model of leadership influence on work-related health, our study results show that leaders’ SelfCare and StaffCare can be applied in a virtual work context. Furthermore, the study revealed that the application of health-oriented leadership is influenced by organizational, social, technical, and personal factors.

### 4.1. Health-Oriented Self-Leadership

Leaders’ profound consciousness about their own health was expressed by a high evaluation of the value of their own health and the perception of their state of health. Almost all leaders experienced their own state of health as positive, but the influence of flexible working conditions on health was perceived differently. Krampitz [53] also found a positive state of health among leaders based on a high level of well-being. Self-determined planning and division of work can have a beneficial influence to health [54,55]. Negative influences of working conditions on the mental health of leaders, such as increased stress, strong time and performance pressure, and increased work demands, were also shown in previous studies [54,55,72]. Work carried out on one’s own initiative close to personal stress limits may indicate the phenomenon of interested self-endangerment [60]. The ability to perceive changes in personal health status in good time was in most cases only learned due to intense workloads over a long period of time. The impact of heavy workloads on health was also shown by a previous qualitative study with virtual leaders [72].

Our findings regarding health-oriented self-leadership behavior coincide with the results of other studies. Virtual leaders named regular physical activity, boundary management, social exchange, and a healthy diet as compensatory activities for the psychological stress at work [72]. Boundary management and the use of occupational health services were also identified as health-promoting behavior for virtual leaders [80]. Beyond that, the present study was able to ascertain further health-oriented self-leadership behavior: sleep, recreational activities, time management, and mental handling of stress. Previous empirical studies on the HoL model showed that leaders serve as role models for employees’ self-leadership and thus indirectly influence employees’ health [62,70,81].

So far, there are only very few studies on the perceived health status, value of health, health awareness, and health behavior of (virtual) leaders. Therefore, future studies should investigate which individual factors such as gender, leadership experience, and health awareness have an influence on the application and perception of health-oriented leadership. As Gregersen et al. [46] point out, socio-demographic characteristics of employees or their own stress experiences can have an influence on the perception of leadership behavior. Furthermore, it remains unclear how health-oriented leadership affects the health status of virtual employees and virtual managers themselves.

### 4.2. Health-Oriented Employee Leadership

The leaders rated the value of the health of their team members just as highly as their own. The majority stated that they are also able to perceive changes in their state of health in a virtual working context. Still, some admitted the fact that physical distance and reduced communication quality aggravate employee-oriented health awareness. Differences concerning the self-image as a leader and the perceived responsibility also became apparent in Echterhoff’s study [72]. Higher requirements placed on leaders of virtual teams, amongst other things due to more intensive relationship design and maintenance, were also illustrated in further studies [17,18,33].

We identified a total of five behaviors as health-oriented employee leadership: trust building activities, health-oriented communication, support in boundary management, implementation of face-to-face meetings, and delegation of decision-making authority and responsibility. The majority of participants considered a relationship of trust to be a fundamental prerequisite for the application of health-oriented leadership in a virtual team. Various studies on leadership in virtual teams were also able to highlight the particular relevance of trust for successful virtual collaboration [10,25,26,35,82,83,84,85]. Nearly all leaders named communication on health-related topics as a central health-oriented leadership behavior. Previous studies had also emphasized the choice of a suitable communication medium in terms of richness for virtual communication [14,16,25,33]. In particular, the (re)design of tasks, emotional support, communication of expectations and offers of help were named as effective ways of dealing with exhausted employees [68]. Most leaders cited support in the boundary management of team members as another health-oriented leadership behavior. Agreements on extended availability, such as balancing and coordinating availability times and supporting work-life balance, were also presented in previous reviews on health-oriented employee leadership [16,80]. Personal meetings were being held at regular periods in all virtual teams and had a high relevance regarding health-oriented employee leadership for most of the interviewees. Previous literature had also emphasized the relevance of personal meetings, e.g., at kick-off events with joint ventures [25,80]. An international survey on virtual teamwork showed that regular face-to-face meetings are the most useful form of communication. They are used especially for conflict resolution, relationship and trust building, and effective and comprehensive communication [13]. Lastly, the delegation of decision-making authority and responsibility was described by several leaders as health-oriented leadership behavior. Delegative leadership concepts are recommended for successful virtual teamwork [15,17,24,25], including shared leadership [6,15,18,26,27,28,29,30]. Furthermore, the qualitative longitudinal study by Rigotti et al. [63] found that health-oriented leadership is conducive to the design of job autonomy and participation in the workplace.

Our research results on health-oriented leadership behavior of virtual leaders are generally consistent with previous studies on virtual leadership. Since previous studies on virtual leadership have not specifically examined the relationship to health, future research should focus more closely on which leadership behavior or concrete leadership styles are health-promoting in the virtual context. Existing research so far only provides indications that a delegative style of leadership is positively related to health-oriented leadership. Further quantitative studies could therefore investigate the relationship between a delegative and health-oriented leadership style and health parameters in analogy to the study by Kranabetter and Niessen [70].

### 4.3. Factors of Influence

Flexible working conditions, structural offers and corporate management and culture were identified as decisive factors influencing the application of health-oriented leadership. Our findings on flexible working conditions and structural offers being supportive and aggravating factors correlates with previous research done in virtual teams. Flexible working conditions were found to facilitate a better adaptation of work to private life [7,8,32] and to have a positive impact on health [37,46,56,86]. Consistent with our findings, other studies indicated that geographical distance and time differences in virtual teams may have negative effects on motivation, collaboration and communication [19,24,32,35,36]. Increased demands of virtual teamwork such as irregular working hours and high workloads can have a psychologically stressful effect and lead to the dissolution of boundaries and interested self-endangerment [2,17,37]. The supporting effect of structural offers such as health offers, further education offers and social events for health-oriented leadership has also been recognized in previous literature [72,80]. According to the current state of research, there are no comparable study results to date on the influence of corporate management and culture on the health-oriented employee leadership in virtual teams.

Our findings of social support by team members or other leaders agree with studies in which social relations promoted virtual cooperation and culture [37,87]. Other studies also showed that different language skills and cultural backgrounds make mutual understanding and building personal relationships in virtual teams more difficult [2,24,32,35,36]. Knieps and Pfaff [88] also indicated that social relationships can be experienced as stressful. In addition to organizational and social factors, digital media were experienced as supportive [17,80]. Previous studies also highlighted the challenges of virtual communication on the basis of technical problems [11,35] and mental stress caused by use of ICT [17,38]. Furthermore, our study results show that personal factors may have a negative influence on the application of health-oriented leadership.

In further quantitative studies, different influencing factors such as occupational support or the internationality and diversity of the virtual team should be investigated as moderating or mediating variables in the relationship between health-oriented leadership and health parameters. Such research findings could provide a better understanding of the framework conditions conducive to the successful application of health-oriented leadership.

## 5. Strengths and Limitations

Our study provides several strengths. The extensive use and growing demand for virtual teamwork in organizations worldwide illustrates the relevance of this new way of working for research and practice [10,11,13]. As there is still much need for research [39,40], our study provides the first empirical findings on the relationship between virtual leadership and health. Overall, the various methods used coincided and were selected in a manner adequate to the subject matter. In line with the PCI interview method, attention was paid to provide sufficient scope for the statements of the interviewees. Furthermore, conducting interviews by telephone enabled a geographical expansion of the sample as well as a research-economic and flexible procedure. A greater anonymity was perceived to be helpful in building trust. Another strength lies in our sample size, considering the exploratory nature of our study. Our 13 conducted interviews were sufficient to reach a theoretical saturation [89].

Yet this study has some limitations. The gender distribution in our sample represents a limitation of our study, as gender-related differences may exist in leadership behavior [90]. The contacted female leaders neither did not respond, cancelled their participation due to lack of time, nor did meet the inclusion criteria despite interest. Future research should therefore include participants of different sexes. Moreover, the survey context of telephone interviews does not allow for standardization. Accordingly, external influences could not be controlled. Thus, a few interviews were technically or personally interrupted—however without consequences for the course of the interview. In addition, socially desirable response behavior could not be entirely avoided. It remains questionable whether the interviewees would attribute the same high relevance to health or would report obstructive leadership behavior outside the interview situation. Furthermore, it remains unclear how the reported health-oriented leadership behavior is perceived and evaluated by team members. Future research must therefore take into account external reports as well as self-reports.

## 6. Practical Implications

Our study results make clear that health-oriented leadership can also be applied in virtual teams. The findings on health-oriented self- and employee leadership provide first helpful insights and recommendations for occupational health promotion in virtual teams. In particular, our results on behavioral patterns and influencing factors regarding the application of health-oriented leadership in virtual teams suggest that organizations can support and relieve managers in health-oriented self- and employee leadership at different levels. Firstly, occupational health prevention should be accorded high strategic relevance and be reflected in the adjustments made to working conditions to ensure the satisfaction and health of employees. Secondly, attention should be paid to the technical functionality of ICT and ergonomic equipment of the workplaces. In addition, regular personal team meetings should also be made financially and organizationally possible in virtual teams to ensure successful and trusting cooperation and thus strengthen social relationships and team cohesion. Further support options should be implemented by expanding structural offers — especially for virtual teams. In particular, leadership-specific further training courses on health-oriented communication and boundary management in virtual teams can have a supporting effect for leaders. Lastly, blended learning training concepts about health-oriented self- and employee leadership are suggested to sensitize all team members about their own health prevention. Basically, it is recommended to adapt the implementation of the measures to contents, team composition and needs of the respective virtual team. Still, it should be pointed out that the working conditions of virtual teams pose specific challenges with regard to the implementation of health-oriented leadership as well as of preventive health measures and therefore have to be considered and supported by organizations.

## 7. Conclusions

Our study was the first to qualitatively assess health-oriented leadership according to the HoL model [64] and to explore it in virtual teams as a new field of application. By using a qualitative research approach, we identified health-oriented behavior patterns and influencing factors concerning its application. For a more comprehensive understanding of health-oriented leadership in virtual teamwork, the consideration of team members in further studies is necessary. This study provides a basis for further qualitative and quantitative studies, in which, among other things, relationships between health-oriented self- and employee leadership and the state of health of team members but also of virtual leaders should be investigated. Based on our findings regarding leadership behavior and influencing factors, practical implementations are presented.

## Figures and Tables

**Table 1:** Contents of the interview guide.

Professional Situation	Characteristics of the Virtual Team	Health-Oriented Leadership in Virtual Teams	Socio-Demographic Data
Profession	Number of teams	Value of health	Gender
Department	Leadership experience	Health awareness	Age
Industry	Team composition	Health behavior	Highest degree
Company size	Geographical distribution	Factors of influence	
Employment	Face-to-face meetings	Improvement proposals	
Working hours	Virtual communication		

**Table 2:** Participants’ characteristics (*n* = 13).

Variable	*n*	%
Gender		
Male	13	100.00
Female	0	0.00
Age		
30–40 years	5	38.46
41–50 years	4	30.76
>50 years	4	30.76
Industry		
IT/Software	8	61.54
Manufacturing	2	15.38
Aerospace	2	15.38
Logistics	1	7.69
Company size		
1–49 employees	0	0.00
50–249 employees	6	46.15
>250 employees	7	53.85
Employment		
Full-time (permanent)	11	84.62
Part-time (permanent)	2	15.38
Main workplace		
Company office	8	61.54
Home-Office	5	38.46
E-Leadership experience		
<2 years	5	38.46
2–4 years	6	46.15
>4 years	2	15.38
Number of team members		
1–5	4	30.76
6–10	5	38.46
11–15	1	7.69
16–20	1	7.69
>20	2	15.38
Geographical distribution		
National	8	61.54
International	5	38.46
Face-to-face meetings		
Yearly	7	53.85
Monthly	3	23.08
Weekly	2	15.38

**Table 3:** Health-oriented leadership behaviors of virtual leaders.

Health Oriented Self-Leadership	Health-Oriented Employee Leadership
Physical activity (10)	Communication (11)
Boundary management (9)	Trust building activities (10)
Nutrition (6)	Support in boundary management (8)
Sleep (4)	Face-to-face meetings (8)
Recreational activities (4)	Delegation (5)
Social exchange (4)	
Time management (3)	
Use of occupational health services (2)	
Mental handling of stress (2)	

The number in brackets corresponds to the number of leaders who have named respective behaviors.

**Table 4:** Factors influencing the application of health-oriented leadership.

Organizational Factors	Social Factors	Technical Factors	Personal Factors
Management board (+/−)	Team (+/−)	Digital media (+/−)	Personal factors (−)
Working conditions (+/−)	Leadership (+/−)		
Corporate culture (+)			
Structural offers (+)			

Supporting factors are marked with (+) and aggravating factors with (−). Influencing factors that were experienced by the interview partners as both supporting and aggravating are marked with (+/−).

## Supplementary Materials

The following are available online at https://www.mdpi.com/1660-4601/17/18/6519/s1, Table S1: COREQ (COnsolidated criteria for REporting Qualitative research) Checklist.

## Appendix A. Interview Guide

Job-related and demographic information will be taken prior to the main interview questions.

1. How important is your health to you—also in the context of digitalized leadership work?
  - How do you experience your health at work?
  - How important is it to you to pay attention to the health of your virtual team members?
2. How do you experience changes in your health condition?
  - Do you have the feeling that you can perceive strain in time?
  - When do you notice changes in your own state of health?
  - What makes it more difficult for you to perceive changes in your own state of health?
  - Is it also possible for you to recognize in virtual teamwork whether the health condition of your team members changes?
3. In which areas do you experience attentive behavior towards yourself?
  - Is it possible for you to lead your virtual team members in a health-oriented manner?
  - What does it mean to you to work healthy and be able to lead health-oriented in a virtual working environment? How do you determine this?
4. What supports or makes it more difficult to lead yourself health-oriented in a virtual working environment?
  - What supports or makes it more difficult to lead your team colleagues health-oriented in a virtual working environment?
  - What influences your health-oriented behavior or efforts in a virtual working environment? Can you give me examples of your everyday business?
5. What suggestions or ideas do you have for the application of health-oriented leadership in virtual teams?
  - What do you wish for to be able to take better care of your own health and that of your team colleagues?
  - What needs to be changed urgently?
